# What we learned from creating one of the world’s most popular MOOCs

**DOI:** 10.1038/s41539-019-0046-0

**Published:** 2019-06-14

**Authors:** Barbara A. Oakley, Terrence J. Sejnowski

**Affiliations:** 1 School of Engineering, Oakland University, Rochester, MI 48309 USA; 2 Howard Hughes Medical Institute, The Salk Institute for Biological Studies, La Jolla, CA 92037 USA; 3 Division of Biological Sciences, University of California, San Diego, La Jolla, CA 92093 USA

**Keywords:** Education, Education

## Abstract

*Learning How to Learn* (LHTL) is currently one of the world’s most popular massive open online course (MOOC), with nearly 2.5 million registered learners in its first 4 years. Here, we “reverse engineer” the design of the course’s videos to show how creative application of well-known principles of multimedia learning in an MOOC context appear to have fueled the course’s popularity. Gaps in knowledge of multimedia learning are also noted. There have been some 50 years of experience researching effective classroom teaching, but less there have been only 5 years since MOOCs became widespread. The success of LHTL may provide further insight into the importance of the principles of multimedia learning, and how those principles might be practically implemented to improve MOOC making and the general design of instructional videos.

## Introduction

Massive open online courses (MOOCs) burst on the scene in 2011 with a major article in the *New York Times*.^1^ The large numbers that enrolled, coupled with the unprecedented reach through the internet, piqued the world’s attention. Just as the internet disrupted the domains of knowledge, commerce, and social networks, it appeared that the world of education was about to change. Almost overnight, new companies were founded to develop and freely distribute lectures online by some of the best educators in the world. These are available on demand anyplace and anytime that there is an internet connection. The latest data available, as of the end of 2018, indicates there are 11,400 extant MOOCs, servicing 101 million learners.^2^

## Background about the MOOC Learning How to Learn

Although many of the most popular MOOCs today involve practical skills such as computer programming or marketing, a few self-help-type courses, such as Yale’s *The Science of Well-Being* and our own *Learning How to Learn* (LHTL) make the top listings. Indeed, LHTL, offered through the Coursera platform, rapidly became and remains one of the world’s most popular online courses, with nearly 2.5 million registered users from over 200 countries worldwide in the 4 years since its initial launch in August 2014. The course continues to attract approximately 1000 new learners each day.

Similar to many MOOCs, the age distribution of LHTL learners has a core in the 25–34-year-old age range—in other words, college students are not the key demographic. Over half of LHTL students have a college education, and many of these have higher educational degrees. Roughly two-thirds of students are male, which is fairly typical for all MOOCs—partly a result of the highly international nature of many English-language MOOCs, which encompass very different cultures. (Only 31% of LHTL students are from North America.)

MOOCs in general, and LHTL in particular, have proven popular with recently graduated students who are in the workforce and in need of new skills. The flexibility of online learning makes it possible for them to fill skill gaps while continuing to work full time (50% of all LHTL students are currently employed). Rapidly advancing technology has made many jobs obsolete while at the same time creating new jobs requiring different skills. MOOCs serve this group well. Adult learning is more difficult when time is short, stresses are high, and the brain might not be as nimble as it once was. LHTL is an enabling MOOC that, by teaching about best practices in learning, may improve learning in other MOOCs. This may account in part for why LHTL has been so popular with a slightly older-than-college-age group, since it helps adult learners, with their often-conflicting employment and family demands, make more efficient use of their learning time.

The LHTL course is organized into four one-week modules, with approximately ten 5-minute videos each week, accompanied by mid-week and end-of-week multiple-choice quizzes, and one end-of-the-course multiple-choice final exam. There are also optional in-video quizzes, peer-reviewed projects, and videos that feature interviews with learners from various walks of life.

The subject matter of LHTL encompasses many of the best insights researchers have discovered about effective learning, including information about topics such as interleaved practice, deliberate practice, and the value of frequent, low-stakes testing.^3,4^ But we also included research insights involving issues such as chunking,^5^ procrastination,^6^ and why exercise^7^ and sleep^8^ are so valuable in the learning process. Where possible, learning concepts are framed within a neuroscientific context. Thus, LHTL begins by describing the difference between the brain’s task positive and task negative (“default mode”) networks, and how these different networks each play a role in our ability to assimilate complex information.^9–11^ The value of sleep in the learning process is described by showing what happens physiologically to neurons in the brain when sleep occurs.^12,13^

The first study involving the effectiveness of the use of LHLT showed promising results.^14^ As the study notes, after the LHTL intervention, “…students of the experimental group reported cramming less, using more learning techniques, redoing exercises with answers covered, and spacing repetitions more often instead of massing. Students’ online study behavior verified their answers to the questionnaire and showed a more efficient use of the learning platforms. Students also benefited from this intervention by scoring higher in the final proficiency test of the second semester.”

It seems that the fresh, multidisciplinary approach to the subject matter of the materials, coupled with the useful nature the insights presented, makes the course attractive for learners. However, we do not believe that it is the subject matter alone of our MOOC that has made it so popular, since other MOOCs related to learning have not been nearly as popular. It is often difficult to find comparative enrollment numbers, but the number of public reviews on a general MOOC review platform can serve as a rough proxy. On Class Central (https://www.class-central.com), one of the most widely used MOOC review platforms, LHTL has 6712 reviews, while the few other more general learning-related courses have far fewer: Athabasca University’s “Learning to Learn Online” has 5 reviews, and Monash University’s “Introduction to Psychology: The Psychology of Learning” and Stanford’s “Reading to Learn in Science” each have 1 (as of March 2019). Popular subjects, such as python programming, machine learning, or happiness, often have a number of competing MOOCs from different universities and online platforms that tackle the subject matter, but it appears that LHTL is so popular in its niche that it has deterred rather than attracted direct competitors.

As described above, if it is not necessarily the subject matter of LHTL that has made the MOOC so popular, what has? A strong remaining option is the presentation and instructional methods. What are those methods? As described in detail below, the methods are, in essence, a practical implementation of Richard Mayer’s well-researched principles of multimedia learning. This means it is possible to unpack some of the essential elements that have helped spur LHTL’s success so they can be more easily replicated in other MOOCs and online courses. We also point to common MOOC production practices (avoided by LHTL), which violate multimedia learning principles. It is worth conjecturing that the violation of multimedia learning principles might hamper the reception of some MOOCs.

## The power of video in MOOC making

“Multimedia learning” is defined by Mayer to mean learning from both pictures and words—multimedia learning has been found to be far more effective than using words alone. As Mayer notes, “Multimedia learning occurs when people build mental representations from words (such as spoken text or printed text) and pictures (such as illustrations, photos, animation, or video)”^15^ (pp. 2–3).

Mayer and his colleagues^15^^[,16^ have developed some 30 principles of multimedia learning that lay out a foundation for how to effectively convey ideas while taking into account the limitations of working memory. (A more readable, if less research-heavy, guide through the principles can be found in Clark and Mayer’s *E-learning and the Science of Instruction*.) These principles can at times seem deceptively simple. The signaling principle, for example, states that “people learn better when cues are added that highlight the organization of the material”—an idea that some (unfortunately not all) instructors might take for granted. But the simplicity is key to the value of the insights. Taken in gestalt, the principles provide an elegant, “reduced-to-the-essence” structure for understanding how and why human beings are able to move the concepts they are learning through their limited working memories and into long-term memory—ultimately to use what they’ve learned in a flexible variety of circumstances. The principles of multimedia learning that are applicable to video are shown in Table 1.

**Table 1:** Mayer’s principles of multimedia learning relevant to video (adapted from Mayer^15^ via Johanes and Lagerstrom^17^)

Principle	Explanation
Multimedia	People learn better from words and pictures than from words alone
Coherence	People learn better when extraneous information is excluded
Signaling	People learn better when cues are added that highlight the key information and its organization
Spatial and temporal contiguity (split attention)	People learn better when words and pictures are physically and temporally integrated
Pre-training	People learn better when provided with pre-training in names and characteristics of key concepts
Segmenting	People learn better when information is presented piecemeal rather than all-at-once
Modality	People learn better from graphics and narration than from graphics and printed text
Personalization	People learn better when words are presented in conversational rather than formal style
Voice	People learn better with a standard-accented voice
Embodiment	People learn better when on-screen agents display humanlike gestures and movements
Animation	People do not necessarily learn better from an animation than from static diagrams
Image principle	People do not necessarily learn better by having the image of an instructor on screen.

According to the extended definition of Mayer’s *modality principle*^3^ (p. 227), “presenting some information in visual mode and other information in auditory mode can expand effective working memory capacity and so reduce the effects of excessive cognitive load [and] substantially increase learning” (see also pp. 316–344). Thus, although multimedia learning in a “distant” (online) format can arise from web pages with text and pictures, and links to e-books and handouts as well as to videos, video can generally provide for a more effective learning experience. Moreover, according to the *spatial* and *temporal contiguity principles* (pp. 279–315), people learn better when words and pictures are presented close to one another in time and space. These contiguity principles can often be more easily accomplished through well-made video than through written text, where the eye must move back and forth between the text and the image.

In related findings from different bodies of research, John Hattie’s immense synthesis of meta-analyses measured the effect of different factors on educational outcomes. Direct instruction was found to be amongst the most effective of all educational interventions^18^ (p. 205.). Many aspects of direct instruction can be conducted using video combined with active online exercises.

Additionally, previous studies have shown that students of, for example, the first course on edX (Circuits and Electronics), spent the majority of their time watching videos,^19,20^ while another study of three Coursera courses found that many students focus on the videos while skipping the other, more interactive components.^21^As de Koning et al.^22^ note in their introductory article in a recent special issue of *Computers in Human Behavior* devoted to instructional video, “instructional video is currently considered one of the most popular ways of delivering instruction.”

In sum then, students tend to focus on videos in online environments, and video provides a powerful mechanism for instructors to take advantage of a number of important multimedia learning principles. Fortunately, modern MOOC platforms lend themselves to video, perhaps even more so than the published text-with-pictures, e-books, or downloadable printed file format. Of course, videos within MOOCs are often accompanied by quizzes, exams, and venues and forums where learners can ask questions and discuss the material, all of which add to the pedagogical value of the MOOC, just as they add value in face-to-face classes.^3,4^

Following these best practices of most of today’s MOOCs, LHTL provides its students with opportunities to interact with one another through discussion forums, and also provides multiple-choice quizzes and projects that are very similar to those of other MOOCs. This begs the question. If the subject matter, testing, projects, and opportunities for interaction of LHTL aren’t necessarily a distinctive draw for students, what is?

## The principles of multimedia and the production of LHTL’s videos

It is important to note that two very different aspects of learning are being referred to in this paper. The first aspect pertains to the subject matter of the LHTL MOOC—that is, the insights about effective learning that are being presented to students in the course. These were described in the introductory section of this paper. But the second aspect relates to LHTL’s presentation and instructional methods as manifested in its videos. In fact, we believe that LHLT differs from other MOOCs primarily in the construction of it videos.

People learn best when the material is presented in a way that captures attention while also channeling information through limited working memory into long-term memory through several sensory channels. These basic ideas, and more, are elucidated in Mayer’s comprehensive set of multimedia principles. We feel LHTL’s effective use of many of Mayer’s multimedia principles, as manifested in the video presentation and instructional methods of LHTL and which distinguish LHTL from many MOOCs, have helped fuel its popularity. But our experience with LHTL also points towards areas of the multimedia principles that might be further explored and developed. The discussion below provides a more nuanced context for these observations.

We divide the discussion into three areas: 1. Use of green screen, 2. Imagery and motion, and 3. Verbiage—and humor. Green screen may seem an oddly practical subheading by comparison with the imagery and verbiage of sections “Background about the MOOC Learning How to Learn” and “The power of video in MOOC-making”. But as we have discovered in the production of LHTL, green screen offers surprising advantages in relation to multimedia learning, so it is worth breaking out separately to begin the discussion.

### Use of green screen

Mayer’s multimedia principles are quite rightly presented as “technologically agnostic.” In other words, the principles apply regardless of the technology being used to convey the information. But one surprising finding we have learned from LHTL is the extraordinary ability of green screen approaches to allow many of Mayer’s multimedia principles to easily be put into play.

**Fig. 1:**
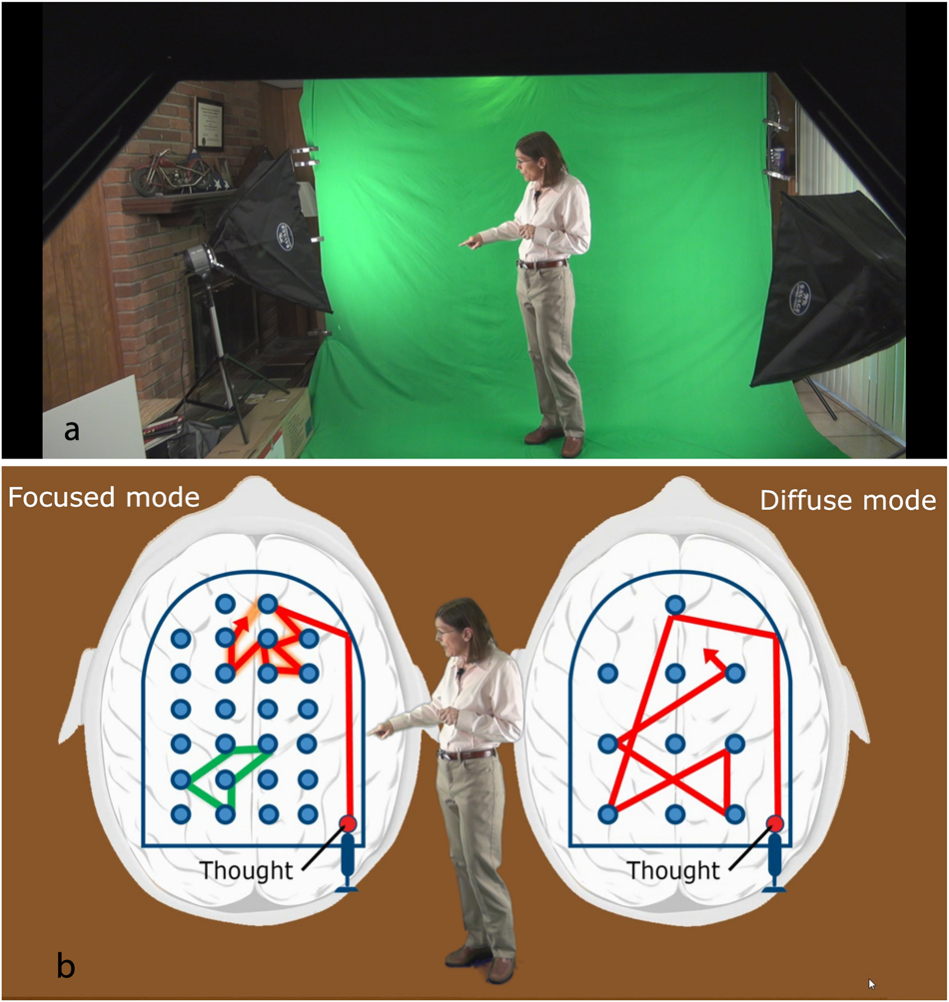
**a** The instructor in the original studio shot and **b** in the final version shown on the video. With green screen, there is less concern about splitting attention between a box containing “talking head” and the illustrations or animations on screen. Not shown here is how the PowerPoint imagery was gradually introduced on the screen in accordance with Mayer’s segmenting and pre-training principles

Green screen involves the ability to integrate an instructor seamlessly into the material being taught. For example, Fig. 1 (top) shows one of the LHTL instructors pointing to the portion of the illustration as it is being described. The lower part of the image shows the original “green screen” version of the video, with the background of the studio (the basement of the instructor’s home), the hood of the teleprompter, and the studio lights. To work with green screen video, one simply crops out the extraneous elements, leaving only the green around the instructor, and the green is eliminated by turning it “transparent” with editing software. Then the entire background is replaced with the desired image or animation. In this case, the desired imagery was a simple PowerPoint slide with animation.

This “instructor inserted into an animation” approach takes advantage of every one of Mayer’s principles (save the animation and image principles) described in Table 1.

It is perhaps best to begin clarifying how the multimedia learning principles apply in this case by contrasting what the video would have been like if the instructor had, instead of being inserted into the video via green screen, been simply inserted as a square box of a talking head in the corner of the screen, as with many online videos. In that case, the *signaling principle* would have been violated in that the instructor would have had difficulty pointing out of the square box into the screen. Even if an arrow had also been added to the PowerPoint, this approach would have violated the *split attention principle* in that there would have been two focal areas on the screen, increasing the cognitive load as the learner attempted to integrate the two separate sources of information.

It is worth noting that one of Mayer’s findings, the *image principle*, states that learning is not improved by having the image of an instructor on screen. One could then reasonably ask, might it be easier to simply indicate salient areas of the PowerPoint animation with an arrow, instead of going through the trouble to film the instructor for insertion into the video? Our sense is that the instructors’ upbeat presences served as friendly guides through the MOOC, even as the instructors’ gesticulations reinforced what was being taught.^23,24^ Simply inserting an arrow into the PowerPoint to direct attention as needed would have been noticeably less personal and, without the animated presence and appropriate gesticulations of the instructor, perhaps less engaging. (One can imagine how students might feel if an “in-class” course were taught via PowerPoint with arrows indicating significant points, but with only the disembodied voice, rather than the physical presence, of the instructor).

In this regard, it is worth noting that the studies behind the *image principle*, summarized in Chapter 14 of *The Cambridge Handbook of Multimedia Learning*, appear to have presented either animations or a “talking head”—they did not seem to involve the green screen presence of an instructor integrated into the context of the material under discussion. More recent research that does involve green screen did not have the instructor interacting in any way with the materials being presented—a “fencepost” approach more akin to a talking head in a box than to the full capabilities of green screen.^25^ (This can be thought of as a violation of the *embodiment principle*, in that on-screen agents, although human, are not displaying humanlike gestures and movements.) Other recent research involves the instructor with a whiteboard somewhat akin to green screen, but examining only brief timeframes rather than an entire course.^26,27^ It might be of value in future research to further examine instructors inserted via green screen to test whether the *image principle* is applicable as presently understood in broader contexts. Thus, future research might involve, not just brief vignettes, but also whether instructor screen presence positively impacted student’s affective learning, cognition, and motivation through an entire course, as previous research has found.^28^ In any case, the green screen approach used to create LHTL was simple and low cost, although time consuming—the bulk of all filming was done in a home basement and the resulting raw footage was edited by one of the co-instructors.

The green screen approach is perhaps one of the best of present video-making techniques in its ability to incorporate the *coherence principle*, whereby extraneous information is excluded. This is because with green screen, anything that is included on the final video must be deliberately selected to be onscreen. This is very unlike many current MOOC videos, where the busy and complex ambient environment—for example, a study with bookcases, pictures, and plants—are often part of the scene presented to learners.

It is worth pointing out that green screen approaches lend themselves to ready use of visual metaphors and analogies, which can more rapidly onboard students onto new ideas.^29^ In fact, metaphors and analogies in teaching may simply be a subset of the *multimedia principle*, with the instructor’s voice, physical presence, and gesticulations^23^ helping the learner to build a mental bridge between an (often visual) concept they are already familiar with, and the new concept. Evidence from neuroscience is confirming that metaphor is, in fact, a useful tool in effective teaching: *neural reuse theory* posits that when learners first understand a concept through the use of metaphor, they are using the same neural circuits that will ultimately be used to understand the new, in-depth topic.^30^ With green screen techniques, students can see the metaphors in an almost live, full-sized form—much as great scientists do in their minds’ eye.^31–33^

On a side note, according to the Merriam-Webster dictionary definition, a *metaphor* is “a figure of speech in which a word or phrase literally denoting one kind of object or idea is used in place of another to suggest a likeness or analogy between them.” An *analogy*, on the other hand, is “a comparison of two otherwise unlike things based on resemblance of a particular aspect.” So there are subtle differences in the usage of the two terms, but both terms convey a sense of similarity between seemingly dissimilar objects or ideas. LHTL, for example, uses an animation of a pinball machine as a metaphor for the function of brain. But elsewhere in the course, the pinball machine is referred to as being “like” the function of the brain—in other words, the pinball machine is used as analogy.

In sum, the green screen approach superbly lends itself to creating video in line with the best principles of multimedia learning: integration of voice with imagery, allowing images to unfold piecemeal even while extraneous information is kept from the viewer, and allowing the instructor to easily signal the salient points at the right time and place while also providing gesticulation and enthusiastic, embodied presence.

### Imagery and motion

The visual system dominates all other sensory systems in humans: A retina projects a million axons into the brain and around 40% of the human cortex is visual. In contrast, there are only 30,000 auditory nerve fibers that activate <10% of the cortex. It is therefore no surprise that imagery forms one of the cornerstones, along with words, of the multimedia principle. In LHTL, imagery was used wherever practicable to allow students to see as well as hear what we were talking about. Care was taken to develop custom illustrations instead of using bland but easier-to-obtain clip art (a violation of the *coherence principle*, which observes that people learn better when extraneous material is excluded rather than included^15^ (p. 279)). Complex imagery that was developed for textbooks was also avoided—this type of imagery can easily result in cognitive overload, a violation of the *segmenting principle*^15^ (p. 317).

At present, instructional designers and instructors themselves naturally tend to think in terms of conventional, static classrooms when they are filming videos for a MOOC. This means that, during the final editing process of video creation, instructors are generally placed in a template fashion on one side of the screen, with visuals or bullet points arising on the other side of the screen. Even when green screen is used, the instructor might be inserted into a corner of the video, staring directly at the camera, hands often down at their sides (the “fencepost approach”) while saying something like “if you look at the red block toward the top of the picture.” As mentioned earlier, this is a clear violation of both the *signaling* and the *embodiment principles*. These static approaches to video editing are easier for editors, but can result in predictable, sometimes difficult-to-follow videos. In contrast, the instructors of LHTL interact with the visuals, turning their face towards them and pointing, as if the visuals were truly beside them—a clear manifestation of the *signaling principle*. Careful pre-planning of both visuals and script were key to dynamic “interactive”—that is, the instructor appearing interact with the onscreen visuals—videos that are sometimes pleasantly surprising while simultaneously being easy-to-follow.

In a somewhat different vein, researchers are beginning to understand at a deep neuroscientific level how motion, and particularly looming motion, attracts and maintains attention.^34,35^ In LHTL, unlike typical MOOCs, great attention is given to the use of motion to emphasize key ideas and, particularly through unexpected motion, to maintain attention on the video.^36–38^ Rarely do more than 30 s pass before some type of motion or pictorial change unfolds. In LHTL, a body might suddenly seem to loom from full body to half body on the screen, or move from left to right, a new image may fly in, or an arrow will move from one area to another. It is not all motion, of course—video editing is an art, not (at least at present), an algorithm.

Curiously, there is little guidance in this regard in the multimedia principles. In this sense, then, might be valuable to explore the potential of a new *predictability principle* for multimedia learning. It is well known that visual change attracts attention, while static scenes can more easily allow attention to wander.^39,40^ As neurobiologist Bernard Baars, the originator of global workspace theory, notes: “The senses are not simple energy transducers, but fast-adapting neuronal networks at multiple levels of input analysis. When a constant stimulus becomes predictable it is no longer consciously perceived.”^41^

It is reasonable to posit that motion and other visual or audio change and surprises might help viewers maintain attention on a video via “bottom-up” attentional processes, allowing them to more easily resist mind wandering without applying conscious effort.^42^ In our experience, a little unpredictability in the use of visuals seems to go a long way in maintaining viewer interest. (Note that what is being proposed here is different from the *animation principle* (“people do not necessarily learn better from an animation than from static diagrams”^15^ (pp. 513–546).) Our recommendations here are also generally in line with Guo et al.’s^43^ recommendations to “[i]ntroduce motion and continuous visual flow…” in their study “How Video Production Affects Student Engagement: An Empirical Study of MOOC Videos.”

High-quality online instruction melds academia, Silicon Valley, and Hollywood. This unique combination can be a good thing for learning. In fact, Daphne Bavelier and Adam Gazzaley and colleagues’ research involving video games has shown that sizzling animated special effects can help produce neural changes, such as those observed in midline frontal theta and long-range theta coherence. These changes can, in turn, have lasting effects on key learning processes such as attentional allocation, resistance to distraction, working memory, and task switching.^44–47^ The future of MOOC making may well involve the integration of standard video-making with video-gaming, so that the next-generation “videos” on a MOOC may involve interactive, video-game like components.

### Verbiage—and humor

It is now worth bringing back to mind the *personalization*, *voice*, and *embodiment principles* mentioned previously: “People learn better when words are spoken in a conversational rather than formal style with a standard-accented voice and when on-screen agents display humanlike gestures and movements.”

This is a seemingly simple set of principles, but in our experience in LHTL, it is easier said than done to speak and gesticulate in a natural, conversational style when standing in front of the camera, particularly when one is also expected to interact with imaginary visuals that will be put in place in the post-production process. So, just as we took great care in pre-planning the visuals, we also took great care in pre-planning the verbiage to be spoken in the videos. We carefully wrote all text and rechecked it to simplify and eliminate pedantic turns of phrase. Ultimately, we found a teleprompter to be an invaluable tool in implementing the *personalization principle*, although turning away from the teleprompter to point something out (*signaling* and *embodiment principles*) and returning one’s gaze to the proper point in the teleprompter text can sometimes be difficult to manage.

Since the humor in LHTL was so carefully pre-planned and often had a verbal component, it is worthwhile to discuss humor in this section. (We should also observe that some of the course’s humor involved imagery.)

In our discussions with fellow educators, we have found the use of humor in MOOCs, not to mention face-to-face classrooms, to be a sometimes surprisingly contentious topic. Some point towards findings in the *Journal of Educational Psychology* by Shannon Harp and Richard Mayer^48^ that humor can be a “seductive detail” that distracts from learning. It should be noted, however, that the 1998 study which drew this conclusion dealt only with written passages that included irrelevant adjuncts—the study did not deal with the video or lecture form. Research involving humor has found mixed results, although there is a general sense that good-hearted relevant humor can lift the viewers’ affect without harming learner engagement.^49,50^ (It is perhaps tangentially relevant here to observe that many observers find Richard Mayer’s presentations about his research findings to be riveting in part because of Mayer’s engaging sense of humor.)

Previous research involving humor has primarily centered around one question—do students learn better with or without humor? In some contexts, especially if the humor allows the instructor to reemphasize a point after the laughter dies down, it is clear that humor can be helpful.^50^ But in light of today’s competitive online environment, another important question for research is “does humor drive students to preferentially select one course over another?” After all, if a prospective student is faced with two choices for a MOOC—one that presents the material in a clear and understandable way, and another that presents the material just as clearly, but with humor and wit, it is reasonable to presume that students might choose the humorous, witty course. Our own experiences indicate that learners appreciate the humor in LHTL, and we feel that it is one of the few contributing aspects of the course’s success that is not predicted by one of Mayer’s multimedia principles. Perhaps, humor is not appropriately considered to be multimedia—or perhaps, in the sense that humor often brings incongruent words and pictures to mind^51^ and seems to be such an important part of providing competitive educational materials in today’s YouTube-oriented world, humor could be a subject of future multimedia research. One hypothetical phrasing for a humor principle to be tested might be “People may not learn a particular item of instruction better with humor, but people may preferentially select multimedia learning that includes humor.” We cannot help but also note that a more complete version of a humor principle would also encompass multi-cultural environments, so that a corollary to the humor principle might be “The more humorous the video, either due to verbiage or imagery, the more it may find appeal in particular cultures, but the less it may appeal to every culture.”

For example, we have found that zombies seem to be an especially appealing metaphor for automated executive functions (habitual actions) in the Western hemisphere and in Europe. But zombies are unappealing, for example, to Chinese viewers, who are used to zombies as being stiff corpses dressed in the official gown of the Qing Dynasty. Despite the unappealing nature of the zombies, however, we should observe that LHTL has proven popular in China. A “for university credit” version of the course, “The Tao of Learning,” (學習之道, literally “The Way to Learn”), with four additional hours of Chinese language video placing the course in a Confucian framework, is available on four of China’s largest MOOC platforms: XueTangX, NetEase, CNMOOC, and eWant. “The Tao of Learning” also won Taiwan’s “Best MOOC” for Fall of 2017.

**Fig. 2:**
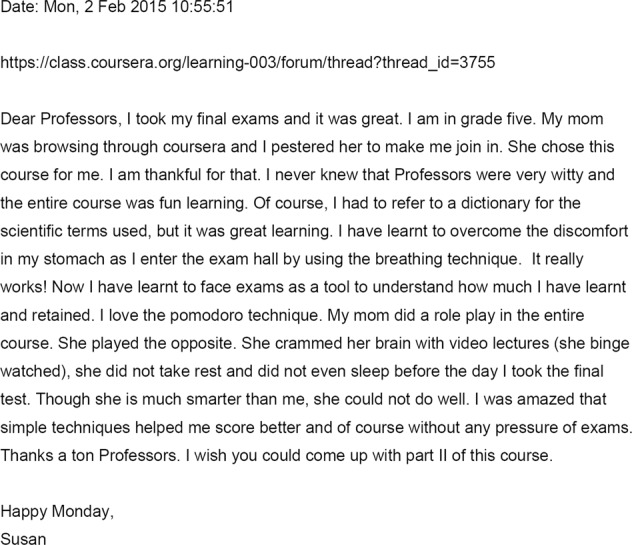
A letter from a young learner in Learning How to Learn (LHTL) that indicates the value of the course’s humor

Humor was not everywhere in LHTL—the course presents very solid and serious research findings. But humor was used as a leavening through the course. As the young fifth-grader commented in Fig. 2, she had never understood before that professors could be witty. Again, since the MOOC was filmed from carefully prepared scripts, it was easy to appear to be spontaneously clever on occasion, although that was sometimes far from the case.

## Conclusions

In drawing back to review what has been learned in the 4 years since the original launch of LHTL, it is clear that although many MOOC students find the content of the course to be valuable, it is the presentation style and instructional methods used in creating the LHTL videos that help set the MOOC apart from many less popular MOOCs. LHTL has successfully applied many useful principles of multimedia learning by creative use of green screen effects coupled with careful pre-planning of both scripts and visuals. The integration of the image of the professors into videos via green screen techniques helped avoid cognitive overload on learners even as it provided for upbeat professorial enthusiasm and encouragement; allowed for appropriate signaling of the salient material at precisely the right time and place; and avoided extraneous information. Care in post-production processes allowed for attention-grabbing motion and other visual or audio change and surprises that may help viewers maintain attention via “bottom-up” attentional processes. In the context of this analysis, we also found several potential gaps in the current set multimedia principles, in particular related to motion, instructor image, and (perhaps) humor.

Ultimately, the observance of many of Mayer’s principles of multimedia learning in the creation of the videos for LHTL appear to have helped underpin LHTL’s success. This provides insight into how future MOOC makers might also integrate multimedia learning principles to improve their MOOC making.
